# Immunomodulatory Effects of Combination Therapy with Bushen Formula plus Entecavir for Chronic Hepatitis B Patients

**DOI:** 10.1155/2019/8983903

**Published:** 2019-01-13

**Authors:** Long-Shan Ji, Qiu-Tian Gao, Ruo-Wen Guo, Xin Zhang, Zhen-Hua Zhou, Zhuo Yu, Xiao-Jun Zhu, Ya-Ting Gao, Xue-Hua Sun, Yue-Qiu Gao, Man Li

**Affiliations:** ^1^Laboratory of Cellular Immunity, Institute of Clinical Immunology, Shanghai Key Laboratory of Traditional Chinese Clinical Medicine, Shuguang Hospital, Affiliated to Shanghai University of Traditional Chinese Medicine, China; ^2^The Pennington School, 112 W Delaware Ave, Pennington, NJ 08534, USA; ^3^Department of Hepatopathy, Shuguang Hospital, Affiliated to Shanghai University of Traditional Chinese Medicine, Shanghai 201203, China

## Abstract

**Aim:**

To compare the clinical efficacy of the combination therapy with Bushen formula (BSF) plus entecavir (ETV) in naïve chronic hepatitis B (CHB) patients and that in CHB patients with partial virological response to ETV and explore the relevant immunoregulatory mechanism.

**Materials and Methods:**

Two hundred and twenty CHB patients were enrolled in the historical prospective cohort study. Patients were categorized into a treatment group (T-Group: combination therapy with BSF plus ETV) and a control group (C-Group: ETV). Patients in T-Group and C-Group were grouped into T1/C1 (treatment-naïve patients) and T2/C2 (patients with partial virological response to ETV). Biochemical assessment, viral load quantitation, and HBV markers were tested. Chinese medicine symptom complex score was evaluated and recorded as well. In addition, peripheral blood mononuclear cells were separated from blood samples in 56 patients and 11 healthy donors. The frequencies of Th1, Treg, and dendritic cells (DCs) and expression levels of PD-1/PD-L1 were examined by flow cytometry.

**Results:**

In treatment-naïve CHB patients, complete viral suppression rates in HBeAg(−) patients were higher than those in HBeAg(+) patients in both T and C groups. In patients with partial virological response to ETV, the rate of HBsAg decline ≥ 20% in HBeAg(+) patients of T2-Group was higher than that in HBeAg(+) patients of C2-Group. A significant reduction of Chinese medicine symptom complex score was only observed in T-Group. The study of mechanism showed that, compared with healthy controls, Th1 and DC frequencies were decreased in all CHB patients, while Treg frequency was increased only in treatment-naïve patients. In addition, compared with healthy controls, PD-1 expression levels on Th1 and Treg were increased in all patients and PD-L1 expression levels on DCs were increased only in treatment-naïve patients. In treatment-naïve patients, the combination therapy with BSF plus ETV increased Th1 and DC frequencies and decreased Treg frequency, which was correlated with HBsAg decline. In addition, in patients with partial virological response to ETV, the combination therapy downregulated PD-L1 levels on DCs and the frequency of Treg, which was related with HBsAg decline.

**Conclusions:**

In patients with partial virological response to ETV, HBeAg(+) patients tend to achieve ideal effects after the combination therapy with BSF plus ETV, which may correlate with the decrease of Treg frequency and the downregulation of PD-L1 levels on DCs.

## 1. Introduction

Hepatitis B is a potentially serious liver disease caused by hepatitis B virus (HBV), which is a general worldwide health problem. According to the reports from WTO, an estimate of 240 million people are persistently infected with HBV and more than 686,000 people die each year due to severe complications caused by hepatitis B, including cirrhosis and liver cancer [1]. The prevalence of hepatitis B is the utmost in sub-Saharan Africa and East Asia. In China, hepatitis B is one of the top 3 infectious diseases reported by the Ministry of Health [2] and 300,000 people die from HBV-related liver diseases each year [3], which holds 40%–50% of the total HBV-related deaths worldwide [4].

Peginterferon and nucleoside/nucleotide analogues are currently approved first-line treatments of chronic HBV infection and have been applied for many years [5, 6]. However, these therapies can hardly cure most chronic hepatitis B (CHB) patients and merely suppress HBV replication. Therefore, most CHB patients have to be treated for a long period of life. The outcome of HBV infection depends on the multifaceted interactions between HBV and the host immune system. Innate and adaptive immunities play a pivotal role in HBV clearance [7, 8]. The efficient suppression of HBV replication depends on a combination of innate immunity, adaptive cellular, and humoral immunities [9]. It has been shown that the cellular immune responses, including CTL, Th1, Treg, and DC, are crucial for governing HBV replication [10]. It was also shown that Th1/Th2 imbalance [11], as well as the increased expression of Treg and Th17 cells, acts as an independent risk factor in CHB patients [12].

There has been an obvious growth in the practice of complementary and alternative medicine in recent years worldwide. As an important complementary and alternative medicine, traditional Chinese medicine (TCM) based on syndrome differentiation has been used widely to treat CHB patients in Asian countries [13, 14]. The theory and practice of immunology and infectious diseases were actually applied in TCM from earlier dynasties. Beginning around 1950, scientific evaluation of herb materials used for treatment of viral hepatitis was accepted in China and Japan. Current studies from Gao et al. showed that the combination therapy with peginterferon plus Chinese herbs exerted combinatorial effects in HBeAg(+) CHB patients [15]. Our previous studies showed that BSF had beneficial effects on CHB patients with mildly elevated ALT (1–2 times ULN) by reducing serum ALT and HBV DNA levels, which was relevant with the decrease of CD4^+^CD25^+^ T cell frequency and the increase of the IFN-*γ* level in CD4^+^ T cells [16]. Entecavir (ETV) is one of first-line antiviral agents for treating CHB patients. However, some patients are likely to exhibit a suboptimal response to ETV. Currently, there are limited data on how to approach these patients. In this study, the anti-HBV efficacies of the combination therapy with BSF plus ETV in naive CHB patients and that in CHB patients with partial virological response to ETV were compared and then the underlying immunoregulatory mechanisms were explored, which will be helpful for clinicians to make effective treatment project for CHB patients.

## 2. Materials and Methods

### 2.1. Subjects

This is a historical prospective cohort study. Two hundred and twenty CHB patients with liver and kidney Yin deficiency and damp-heat syndrome were enrolled in this study. The inclusion criteria were patients enrolled for antiviral therapy [17], ETV monotherapy, or combination therapy with BSF plus ETV applied. The normal control group comprised 11 healthy donors who had no evidence of exposure to HBV (HBV surface antigen- (HBsAg-) negative, anti-HBc-negative). All subjects were given written informed consent before the blood samples were collected. This study was conducted in accordance with the ethics principles of the Declaration of Helsinki and regulation of clinical trial. This study was approved by the IRB of Shuguang Hospital affiliated to Shanghai University of TCM.

### 2.2. Drugs and Grouping

According to the treatment project, 220 patients were categorized into treatment-naïve patients and patients with partial virological response to ETV. Then, treatment-naïve patients were categorized into T1-Group and C1-Group, and patients with partial response to ETV were categorized into T2-Group and C2-Group. Patients in T1-Group and T2-Group were administrated with BSF combined with ETV, and patients in C1-Group and C2-Group were administrated with ETV. The above patients were followed up for 6 months. Eleven healthy controls were enrolled as a healthy control group. Peripheral blood mononuclear cells (PBMCs) were separated from blood of 56 patients and 11 healthy controls. The baseline characteristics of 220 CHB patients are shown in Table 1. There were significant differences among four groups in basic levels including ALT, AST, HBV DNA, HBsAg, and HBeAg.

### 2.3. Drugs

The BSF granule (oral agents, one dose) [16] was prepared and provided by Jiangyin River Pharmaceutical Company Limited and included Astragalus mongholicus, Fructus Ligustri Lucidi, Longspur epimedium, ClawVine, Rhizoma Picrorhizae, and Pericarpium citri reticulatae viride, and the manufacturing procedure involved boiling, filtering, plastering, and drying. Patients were given 0.5 mg/day ETV and 4.5 g/day BSF granule, oral administration.

### 2.4. Serum Viral Load HBV Serum Markers and ALT/AST Determination

Serum HBV DNA levels in CHB patients were tested with real-time PCR using a LightCycler PCR system (FQD-33A) with a lower limit of approximately 1000 viral genome copies/ml. The results were considered abnormal when HBV DNA was >1000 copies/ml. Serum HBsAg and HBeAg levels were detected by ELISA with commercially available kits (Sino-American Biotechnology Company).

The serum ALT/AST levels were assayed by DXC 800 Fully Auto Biochemistry Analyzer, at the Department of Clinical Laboratory, Shuguang Hospital affiliated to Shanghai University of TCM, China. The results were considered abnormal when ALT was >40 U/l.

### 2.5. Isolation of PBMCs

PBMCs were isolated from heparinized blood by standard density gradient centrifugation with Lympholyte-H (Cedarlane) according to manufacturer's protocol. The cell viability was over 90%, as assessed by the trypan blue exclusion test.

### 2.6. Flow Cytometry (FCM)

The CD8-FITC, IFN-*γ*-APC-Cy7, CD4-FITC, CD25-APC, FoxP3-PE, lin1-FITC, CD11c-APC, HLA-DR-PE, PD-1-PE-cy7, and PD-L1-PE-cy7 mAbs were obtained from BioLegend (BioLegend, CA). Fix and Perm kit was obtained from BioLegend (BioLegend, CA). Intracellular cytokine production was detected by four-color flow cytometry [16, 18], as described in previous papers. What need to be illustrated is that PMA-ionomycin stimulation causes the prominent alternation of cell membrane expression of CD4, so CD3^+^CD8^−^ cells are identified as CD4^+^ T cells [19]. After washing with PBS, the stained cells were analyzed by flow cytometry.

### 2.7. Symptom Scores

According to our previous paper [20], symptoms in CHB patients were recorded before and after treatment including hypodynamia, shortness of breath, sweating, palpitations, poor appetite, insomnia, characteristics of urine and stools, appearance of tongue, and characteristics of pulse. Then, Chinese medicine symptom complex score was calculated by recording the severity of the symptoms: none, light, moderate, and severe were scored as 0, 1, 2, and 3, respectively.

### 2.8. Statistical Analysis

Statistical analyses were performed using SPSS statistics software (version 21.0, IBM Inc., NY, USA). Nonparametric test of rank transformation was used to compare the differences of intergroups and intragroups. The measurement data and numeration data were statistically analyzed with the *t*-test and Fisher exact tests *χ*^2^, respectively. All statistical tests were a two-sided test; *P* < 0.05, and the difference was statistically significant.

## 3. Results

### 3.1. The Clinical Effects of the Combination Therapy with BSF plus ETV in CHB Patients

**Figure 1:**
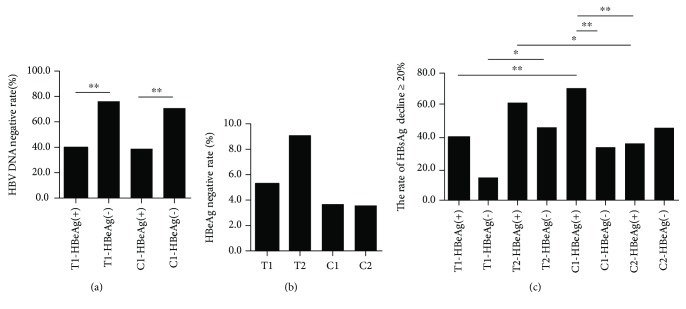
The clinical effects of the combination therapy with BSF plus ETV in CHB patients. Serum HBV DNA, HBsAg, and HBeAg levels were evaluated before and after treatment. (a) HBV DNA-negative rates in HBeAg(−) patients were higher than those in HBeAg(+) patients in both T1-Group and C1-Group. (b) HBeAg-negative rate in T2-Group was higher than that in C2-Group, although there was no significant difference. (c) The rate of HBsAg decline ≥ 20% in HBeAg(+) patients in T2-Group was higher than that in HBeAg(+) patients in C2-Group.

In treatment-naïve patients, the HBV DNA-negative rates in HBeAg(−) patients were markedly higher than those in HBeAg(+) patients in both T and C groups (Figure 1(a)). In patients with partial virological response to ETV, HBeAg-negative rate in T2-Group was higher than that in C2-Group, although there was no significant difference (Figure 1(b)), and the rate of HBsAg decline ≥ 20% in HBeAg(+) patients of T2-Group was higher than that in HBeAg(+) patients of C2-Group (Figure 1(c)). The above results reminded us that the combination therapy with BSF plus ETV may be a potential effective therapy for patients with partial response to ETV.

### 3.2. The Combination Therapy with BSF plus ETV Improved Chinese Medicine Symptoms in CHB Patients

Chinese medicine symptom complex score is the conventional method for assessing the adjustment of Chinese medicine symptoms. Damp-heat stasis syndrome and liver and kidney Yin deficiency syndrome, classified as excess syndrome and deficiency syndrome, respectively, are the common syndromes in CHB patients [21]. The standard for Chinese medicine symptom complex score is based on guiding principles for clinical research on new drugs of TCM. In this study, our results showed that Chinese medicine symptom complex score in CHB patients was decreased from 14.57 ± 4.83 to 8.45 ± 1.09 after treatment in T-Group and there was no significant difference after treatment in C-group, as shown in Table 2, which suggested that BSF substantially improved Chinese medicine symptoms in CHB patients.

### 3.3. Characteristics of Peripheral Th1, Treg, and DCs in CHB Patients

**Figure 2:**
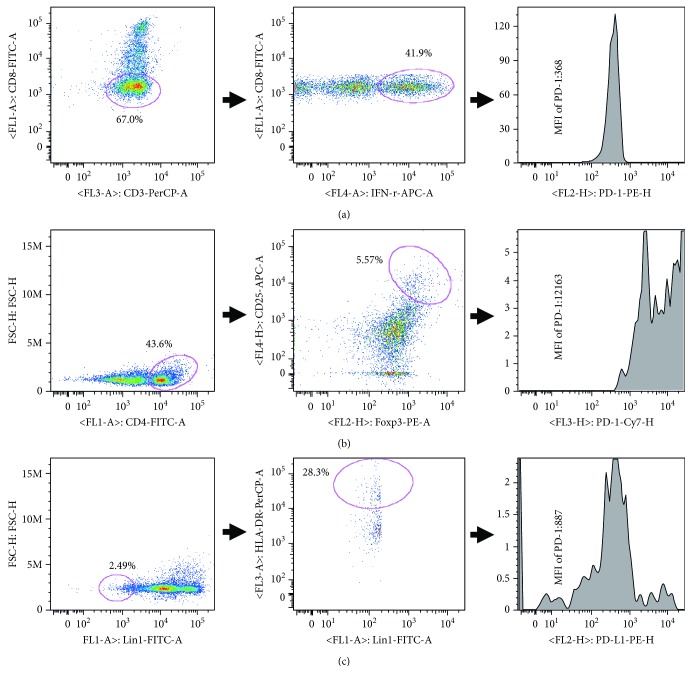
Phenotypic characterization of circulating Th1, Treg, and DCs and expression levels of PD-1 and PD-L1 on immune cells in CHB patients. This panel is based on surface markers CD3, CD8, IFN-*γ*, CD4, CD25, Foxp3, LIN1, HLA-DR, PD-1, and PD-L1. All cells were gated using a FSC-H/SSC-A dot plot. (a) Among PBMCs, CD3^+^CD8^−^ cells were defined as CD4^+^ T cells. Among CD4^+^ T cells, CD4^+^IFN-*γ*^+^ cells were Th1 and then MFI of PD-1 expression on Th1 was analyzed. (b) Among PBMCs, CD4^+^ cells were gated and then CD4^+^CD25^+^FoxP3^+^ cells were defined as Treg. MFI of PD-1 expression on Treg was analyzed. (c) Among PBMCs, LIN1^−^ cells were gated and then LIN1^−^HLA-DR^+^ cells were defined as DCs. MFI of PD-L1 expression on DCs was analyzed.

PBMCs from 11 healthy controls, 32 treatment-naïve patients, and 24 patients with partial virological response to ETV were separated, and the frequencies of Th1, Treg, and DCs were analyzed by flow cytometry. Baseline characteristics of the above subjects are shown in Tables 3 and 4. Phenotypic characterization of circulating Th1, Treg, and DCs and expression levels of PD-1 and PD-L1 on immune cells in CHB patients are shown in Figure 2.

Results showed that the frequencies of CD4^+^ T cells, Th1, and DCs in all patients were markedly lower than those in healthy controls (*P* < 0.01) and the frequencies of CD4^+^ T cells and DCs in patients with partial virological response to ETV were obviously higher than those in treatment-naïve patients (*P* < 0.05 or *P* < 0.01). In addition, the Treg frequency in treatment-naïve patients was higher than that in the normal group (*P* < 0.05) and the Treg frequency in patients with partial virological response to ETV was significantly lower than that in treatment-naïve patients (*P* < 0.01), as shown in Table 5.

PD-1 expression levels on Th1 and Treg in all patients were notably higher than those in normal controls (*P* < 0.05 or *P* < 0.01), and PD-1 levels on Th1 in patients with partial virological response to ETV were higher than those in treatment-naïve patients (*P* < 0.05). PD-L1 levels on DCs in treatment-naïve patients were markedly higher than those in normal controls (*P* < 0.01) as shown in Table 5.

### 3.4. Correlation between the Changes of Immune Cell Subsets and HBsAg Decline after the Combination Therapy with BSF plus ETV

The clinical effects of the combination therapy on CHB patients were analyzed, and the results showed that there was no difference in the viral response rate between T1-Group and C1-Group. However, the rate of HBsAg decline ≥ 10% in T1-Group was higher than that in C1-Group, although the difference was not statistically significant (*P* > 0.05), as shown in Table 5.

**Figure 3:**
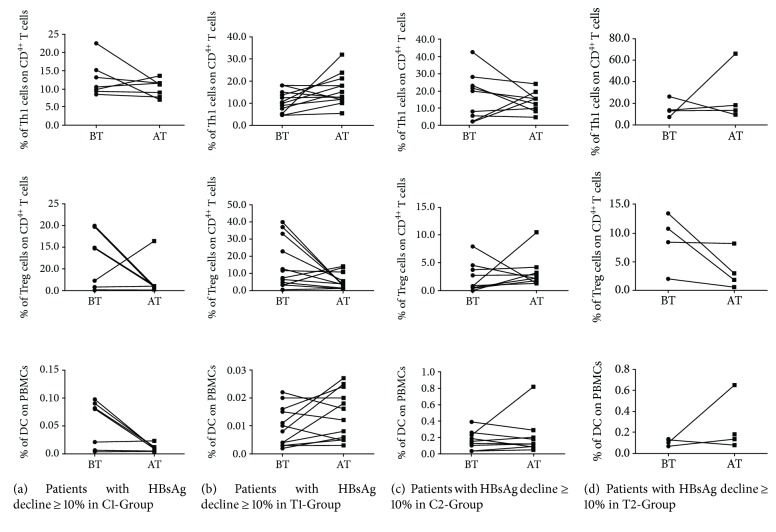
Relationship between the changes of frequencies of Th1, Treg, and DCs and HBsAg decline. (a) Changes of frequencies of Th1, Treg, and DCs were analyzed before and after treatment in patients with HBsAg decline ≥ 10% of C1-Group. BT: before treatment; AT: after treatment. (b) Changes of frequencies of Th1, Treg, and DCs were analyzed before and after treatment in patients with HBsAg decline ≥ 10% of T1-Group. (c) Changes of frequencies of Th1, Treg, and DCs were analyzed before and after treatment in patients with HBsAg decline ≥ 10% of C2-Group. (d) Changes of frequencies of Th1, Treg, and DCs were analyzed before and after treatment in patients with HBsAg decline ≥ 10% of T2-Group.

To explore the immunological mechanism of the combination therapy for treating CHB, the relationships between the changes of frequencies of Th1, Treg, and DCs and HBsAg decline were analyzed. In treatment-naive patients with HBsAg decline ≥ 10%, Th1 and DC frequencies were increased and Treg frequency was decreased in T1-Group, but Th1 and DC frequencies were decreased in C1-Group (Figures 3(a) and 3(b)). In patients with partial virological response to ETV with HBsAg decline ≥ 10%, Treg frequency was decreased in T2-Group, but Treg frequency was not changed and Th1 frequency was decreased in C2-Group (Figures 3(c) and 3(d)). The above results showed that the combination therapy with BSF plus ETV contributed to the increase of Th1 and DC frequencies and the decrease of Treg frequency in treatment-naïve patients and the therapy contributed to the decrease of Treg in patients with partial virological response to ETV. Therefore, the mechanism of the combination therapy with BSF plus ETV downregulating HBsAg levels may lie in regulating frequencies of immune cell subsets.

**Figure 4:**
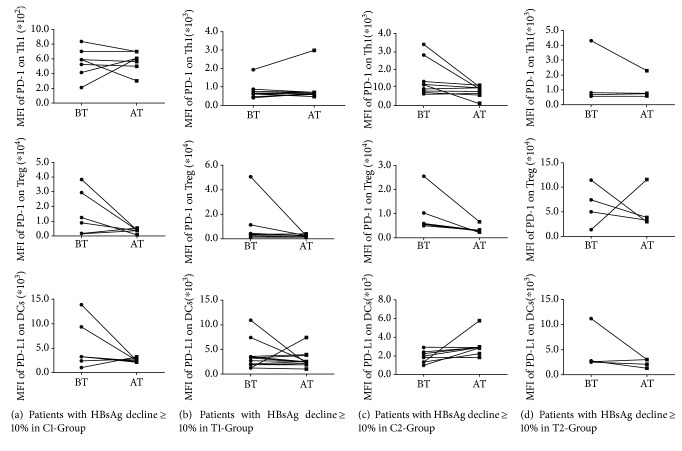
Relationship between PD-1 and PD-L1 levels on immune cell subsets and HBsAg decline. (a) Changes of PD-1 levels on Th1 and Treg and the PD-L1 level on DCs were analyzed before and after treatment in patients with HBsAg decline ≥ 10% of C1-Group. BT: before treatment; AT: after treatment. (b) Changes of PD-1 levels on Th1 and Treg and the PD-L1 level on DCs were analyzed before and after treatment in patients with HBsAg decline ≥ 10% of T1-Group. (c) Changes of PD-1 levels on Th1 and Treg and the PD-L1 level on DCs were analyzed before and after treatment in patients with HBsAg decline ≥ 10% of C2-Group. (d) Changes of PD-1 levels on Th1 and Treg and the PD-L1 level on DCs were analyzed before and after treatment in patients with HBsAg decline ≥ 10% of T2-Group.

In addition, the correlations between PD-1 and PD-L1 expression levels on immune cell subsets and HBsAg decline were analyzed. In treatment-naive patients with HBsAg decline ≥ 10%, the PD-1 level on Treg and the PD-L1 level on DCs were decreased both in C1-Group and T1-Group (Figures 4(a) and 4(b)). In patients with partial virological response to ETV with HBsAg ≥ 10%, PD-1 levels on Th1 and Treg were decreased in C2-Group and the PD-L1 level on DCs was decreased in T2-Group (Figures 4(c) and 4(d)). The above results showed that the decrease of PD-L1 on DCs may be correlated with HBsAg decline in patients with partial virological response to ETV after the combination therapy.

## 4. Discussion

According to current guidelines, the goal of therapy for CHB is to improve quality of life and survival by preventing the progression of the disease to cirrhosis, decompensated cirrhosis, end-stage liver disease, HCC, and death and ETV and tenofovir can be confidently used as first-line monotherapies [6, 17]. However, there are more and more patients who experienced treatment failure to different nucleotide analogue treatment regimens. So what should they do? This is a growing problem in daily clinical practice. One meta-analysis showed that Kushenin combined with nucleotide analogues increased the frequency of loss of serum HBeAg, HBeAg seroconversion, undetectable HBV-DNA levels, and ALT normalization compared with nucleotide analogues [22]. One recent study showed that the liver histological response rate in the combination treatment group with Diwu Yanggan capsule plus ETV was significantly higher than that in the group with ETV (71.43% versus 22.22%; *P* = 0.036) after 48 weeks of treatment [23]. In our study, the clinical effects of combination therapy with BSF plus ETV on CHB patients were observed. The results showed that the combination therapy contributed to the decrease of HBsAg in patients with partial response to ETV, which gave us a hint that the patients who experienced treatment failure to different nucleotide analogue treatment regimens may be administrated with the combination therapy.

According to the TCM symptoms of CHB patients, liver-gallbladder damp-heat and liver stagnation-spleen deficiency are the most abundant syndromes [21]. BSF was composed of Astragalus mongholicus, Fructus Ligustri Lucidi, Longspur epimedium, ClawVine, Rhizoma Picrorhizae, and Pericarpium citri reticulatae viride, which was used to tonify kidney, invigorate spleen, and remove dampness. Our results showed that Chinese medicine symptom complex score in CHB patients was significantly decreased after the combination therapy. In addition, the rate of HBsAg decline ≥ 20% in HBeAg(+) patients of T2-Group was higher than that in HBeAg(+) patients of C2-Group, which suggested that the combination therapy with BSF plus ETV contributed to the decrease of HBsAg, especially in patients with partial response to ETV. So, the combination therapy with BSF plus ETV will provide a potential treatment method for patients with partial response to ETV.

It has been shown that the components of BSF have a broad-spectrum immunoregulatory effects. Astragalus polysaccharides might suppress Treg activity by binding TLR4 on Treg and trigger a shift of Th2 to Th1 with the activation of CD4^+^ T cells in burned mice with P. aeruginosa infection [24]. The supercritical fluid extract of Fructus Ligustri Lucidi regulated the expression of Th1- and Th2-related cytokines, elevated the levels of IL-2, IFN-*γ*, and TNF-*α* produced by Th1 lymphocytes, and decreased the levels of IL-4 and IL-10 produced by Th2 lymphocytes [25]. Our results showed that the combination therapy with BSF plus ETV increased Th1 and DC frequencies in treatment-naïve patients, which was correlated with the decrease of HBsAg levels. Therefore, Astragalus polysaccharides and Fructus Ligustri Lucidi may play a key role in contributing to the increase of Th1 frequencies. In future studies, we will explore in depth the immunoregulatory mechanism of BSF in treating CHB patients by collecting more samples from patients.

## Figures and Tables

**Table 1:** Baseline characteristics of 220 CHB patients [*M*(*Q*1 − *Q*3)].

Group (*n*)	Sex (M/F)	Age (years)	ALT (U/l)	AST (U/l)	HBV DNA (lgIU/ml)	HBsAg (lgIU/ml)	HBeAg(+) (*n*)
T1 (59)	38/21	37.0 (32.0–42.0)	40.0 (26.0–73.0)	33.0 (26.0–60.0)	5.48 (4.31–7.05)	3.64 (3.22–4.12)	38
T2 (42)	26/16	41.5^a^ (37.3–46.8)	22.0^b^ (14.5–28.2)	24.0^e^ (19.0–30.5)	N.A.	3.37 (2.92–4.00)	18
C1 (78)	59/19	34.0 (29.0–41.0)	53.0^c^ (34.0.0–93.0)	42.0^f^ (29.0–65.0)	7.11 (5.17–7.95)	3.99 (3.34–4.16)	54
C2 (41)	36/5	39.0 (33.0–52.5)	25.0^d^ (22.0–28.8)	25.0^g^ (22.0–28.8)	N.A.	3.56 (3.10–3.80)	19

Notes: M: male; F: female; ALT: alanine aminotransferase; AST: aspartate transaminase; HBV: hepatitis B virus; HBsAg: hepatitis B surface antigen; HBeAg: hepatitis B e antigen. ^a^*P* < 0.05, compared with C1; ^b^*P* < 0.05, compared with T1; ^c^*P* < 0.05, compared with T1; ^d^*P* < 0.05, compared with C1; ^e^*P* < 0.05, compared with T1; ^f^*P* < 0.05, compared with T1; ^g^*P* < 0.05, compared with C1.

**Table 2:** Changes of Chinese medicine symptom complex score in CHB patients (*x* ± *s*).

Group	Case	Before treatment	After treatment
T-Group	101	14.57 ± 4.83	8.45 ± 1.09^∗^
C-Group	119	14.90 ± 4.25	12.38 ± 2.50

Note: ^∗^*P* < 0.05, compared with that before treatment

**Table 3:** Baseline characteristics of CHB patients and normal controls [*M*(*Q*1 − *Q*3)].

Group	Sex		Age (years)	ALT (U/l)	HBV DNA (log10IU/ml)	HBeAg (log10COI)	HBsAg (log10IU/ml)
	M	F					
Normal controls	6	5	25.0 (25.0–26.0)	17.5 (10.6–35.8)	N.A.	N.A.	N.A.
Treatment-naïve patients	26	6	41.0 (35.3–48.5)	57.5 (41.5–96.0)	6.63 (5.13–7.48)	2.28 (0.00–2.88)	3.85 (3.31–4.01)
Patients with partial virological response to ETV	21	3	32.0 (28.3–37.5)	34.0 (23.3–47.0)	N.A.	1.98 (0.23–3.09)	3.85 (3.41–4.14)

**Table 4:** Comparative analysis of peripheral Th1, Treg, and DCs between healthy controls and CHB patients [*M*(*Q*1 − *Q*3)].

	Healthy controls	Treatment-naïve patients	Patients with partial response to ETV
CD4^+^ T cell of PBMCs (%)	60.30 (56.50–64.60)	16.20^∗∗^ (7.30–56.40)	49.30^∗∗^^,△^ (46.00–63.30)
Th1 of CD4^+^ T cells (%)	60.40 (58.80–62.2)	10.50^∗∗^ (8.70–14.1)	10.00^∗∗^ (3.10–14.20)
Treg of CD4^+^ T cells (%)	4.00 (3.09–5.18)	12.68^∗^ (4.98–41.48)	2.88^△△^ (0.50–8.32)
DCs of PBMCs (%)	0.29 (0.27–0.40)	0.07^∗∗^ (0.01–0.13)	0.15^∗∗^^,△△^ (0.10–0.22)
PD-1 on Th1 (MFI)	437.5 (412.8–452.8)	523.1^∗^ (423.2–639.4)	594.5^∗∗^^,△△^ (553.0–696.4)
PD-1 on Treg (MFI)	1118.2 (856.8–2628.9)	7164.1^∗∗^ (3022.4–25690.6)	5677.8^∗^ (1372.3–11151.0)
PD-L1 on DCs (MFI)	2010.30 (1683.5–2852.5)	3432.1^∗∗^ (2080.6–6800.3)	2530.3 (1764.3–4098.9)

Notes: ^∗^*P* < 0.05, compared with healthy controls; ^∗∗^*P* < 0.01, compared with healthy controls; ^△^*P* < 0.05, compared with treatment-naïve patients; ^△△^*P* < 0.01, compared with treatment-naïve patients.

**Table 5:** Clinical effects of the combination therapy with ETV plus BSF.

Group	Case	Sex (F/M)	Age (years) [*M*(*Q*1 − *Q*3)]	Rates of virological response (%)	Rates of HBsAg decline ≥ 10% (%)
T1-Group	18	14/4	46.0 (36.5–49.5)	94.4	66.7
T2-Group	7	5/2	32.0 (29.0–36.0)	/	57.1
C1-Group	14	12/2	43.0 (37.5–49.5)	85.7	50.0
C2-Group	17	16/1	32.0 (27.0–42.0)	/	52.9

## Data Availability

Data are made available to all interested researchers upon request.
