# Corrigendum to “Immunomodulatory Effects of Combination Therapy with Bushen Formula plus Entecavir for Chronic Hepatitis B Patients”

**DOI:** 10.1155/2022/7931597

**Published:** 2022-01-25

**Authors:** Long-Shan Ji, Qiu-Tian Gao, Ruo-Wen Guo, Xin Zhang, Zhen-Hua Zhou, Zhuo Yu, XiaoJun Zhu, Ya-Ting Gao, Xue-Hua Sun, Yue-Qiu Gao, Man Li

**Affiliations:** ^1^Laboratory of Cellular Immunity, Institute of Clinical Immunology, Shanghai Key Laboratory of Traditional Chinese Clinical Medicine, Shuguang Hospital, Affiliated to Shanghai University of Traditional Chinese Medicine, China; ^2^The Pennington School, 112 W Delaware Ave, Pennington, NJ 08534, USA; ^3^Department of Hepatopathy, Shuguang Hospital, Affiliated to Shanghai University of Traditional Chinese Medicine, Shanghai 201203, China

In the article titled “Immunomodulatory Effects of Combination Therapy with Bushen Formula plus Entecavir for Chronic Hepatitis B Patients” [1], an Authors' Contributions section should be added as follows: Long-Shan Ji, Qiu-Tian Gao, and Ruo-Wen Guo contributed equally to this work.

In addition, Man Li, Yue-Qiu Gao, and Xue-Hua Sun should be listed as corresponding authors.
